# Compositional Stability of the Bacterial Community in a Climate-Sensitive Sub-Arctic Peatland

**DOI:** 10.3389/fmicb.2017.00317

**Published:** 2017-03-07

**Authors:** James T. Weedon, George A. Kowalchuk, Rien Aerts, Stef Freriks, Wilfred F. M. Röling, Peter M. van Bodegom

**Affiliations:** ^1^Department of Ecological Science, Vrije Universiteit AmsterdamAmsterdam, Netherlands; ^2^Research Group of Plant and Vegetation Ecology, Department of Biology, University of AntwerpAntwerp, Belgium; ^3^Ecology and Biodiversity, Department of Biology, Utrecht UniversityUtrecht, Netherlands; ^4^Department of Molecular Cell Physiology, Vrije Universiteit AmsterdamAmsterdam, Netherlands; ^5^Institute of Environmental Sciences, Leiden UniversityLeiden, Netherlands

**Keywords:** peatlands, bacteria, climate change, soil organic carbon, soil nitrogen, seasonality, 16S RNA, 16S DNA

## Abstract

The climate sensitivity of microbe-mediated soil processes such as carbon and nitrogen cycling offers an interesting case for evaluating the corresponding sensitivity of microbial community composition to environmental change. Better understanding of the degree of linkage between functional and compositional stability would contribute to ongoing efforts to build mechanistic models aiming at predicting rates of microbe-mediated processes. We used an amplicon sequencing approach to test if previously observed large effects of experimental soil warming on C and N cycle fluxes (50–100% increases) in a sub-arctic *Sphagnum* peatland were reflected in changes in the composition of the soil bacterial community. We found that treatments that previously induced changes to fluxes did not associate with changes in the phylogenetic composition of the soil bacterial community. For both DNA- and RNA-based analyses, variation in bacterial communities could be explained by the hierarchy: spatial variation (12–15% of variance explained) > temporal variation (7–11%) > climate treatment (4–9%). We conclude that the bacterial community in this environment is stable under changing conditions, despite the previously observed sensitivity of process rates—evidence that microbe-mediated soil processes can alter without concomitant changes in bacterial communities. We propose that progress in linking soil microbial communities to ecosystem processes can be advanced by further investigating the relative importance of community composition effects versus physico-chemical factors in controlling biogeochemical process rates in different contexts.

## Introduction

Underlying many studies in microbial ecology is the premise that there is a relation between the microbial community composition (“who is there?”) and the functional potential (“what can they do or are doing?”) of a community (Raes and Bork, 2008; Vandenkoornhuyse et al., 2010). Within the soil environment, many important biological processes are carried out by microorganisms, leading to important ecosystem-level consequences: (i) they can influence the diversity and productivity of plant communities (Van der Heijden et al., 2008); (ii) they mediate important transformations in the cycles of nitrogen, phosphorus, and other nutrients (Falkowski et al., 2008); and (iii) through their activity as decomposers regulate the formation and persistence of soil organic carbon, a globally important carbon store (Six et al., 2002). A primary motivation for soil molecular microbial ecology is therefore the promise of gaining insights into the biological mechanisms underlying these important ecosystem processes.

For microbial communities to be predictive of ecosystem functions, there should be a detectable causative or correlative link between the composition of the microbial community at a given environment, and a (qualitative or quantitative) measure of the ecosystem process of interest (Schimel and Gulledge, 1998; Allison and Martiny, 2008; Amend et al., 2016). Although variation in soil microbial communities have been shown to associate with gradients in, e.g., soil pH (Lauber et al., 2009) or nutrient availability (Fierer et al., 2012), the extent to which such variation in community structure has consequences for biogeochemical processes still remains an open question (Prosser, 2012; Schimel and Schaeffer, 2012; Graham et al., 2014, 2016; Bier et al., 2015). For some transformations, performed by well-defined functional groups, it is possible to make a direct link between community composition and biogeochemical fluxes (e.g., ammonia oxidation, denitrification, methane oxidation; Mertens et al., 2009; Salles et al., 2009; Bodelier et al., 2012; Bier et al., 2015). However, evidence for such a link remains elusive for ecosystem processes mediated by a broader range of microorganisms, such as soil heterotrophic respiration, and the turnover of organic nitrogen. Given the importance of these “general community” processes for understanding and predicting ecosystem dynamics, it is therefore imperative to establish if and when such a link exists.

One of the main potential applications of links between composition and process is the development of Earth system models that explicitly model microbial processes in order to improve predictions of biogeochemical cycling as influenced by global change phenomena (Todd-Brown et al., 2012; Wieder et al., 2015). In this context, a long-term climate manipulation experiment in a sub-arctic peat bog in Abisko, Sweden (Dorrepaal et al., 2004), offers a particularly relevant system to investigate whether perturbations that lead to variation in biogeochemical functions are also associated with changes in the phylogenetic composition of the bacterial community. In this system, experimental climate manipulations have led to persistent increases of approximately 50–100% in carbon and nitrogen cycling rates (Dorrepaal et al., 2009; Weedon et al., 2012). This result has significant implications for terrestrial feedbacks to climate change due to the major global importance of northern peatlands as a long-term sink for atmospheric carbon (Limpens et al., 2008). Given the high climate-sensitivity of peat C and N dynamics at this experimental site, we hypothesized that, if bacterial community composition is indeed linked to general biogeochemical fluxes, then climate treatments that lead to changes in biogeochemical fluxes should also lead to changes in the composition of the soil bacterial community. Conversely, if there has been no change in community composition, then changes in C and N dynamics can be attributed to altered rates of activity of a stable bacterial community, or control by other groups of soil organisms. We used Illumina sequencing of amplicons generated from the V3 region of bacterial 16S rRNA genes and rRNA (Bartram et al., 2011; Caporaso et al., 2012) to describe the bacterial community composition in the peatland climate experiment at Abisko. The simultaneous analysis of patterns in DNA and RNA allowed comparison of patterns between the total (DNA) and potentially active (RNA) bacterial communities (Urich et al., 2008; Baldrian et al., 2012), between which there may be important differences in sensitivity to environmental conditions. Our primary aim was to determine if the previously observed climate effects on peatland C and N cycles also lead to changes to the bacterial phylogenetic DNA and/or rRNA community composition.

## Materials and Methods

### Field Site and Sampling

Sampling for the soil bacterial community was conducted in permanent plots established for a long-term climate manipulation experiment close to the Abisko Scientific Research Station in Abisko, sub-arctic Sweden (68°21′N, 18°49′E, altitude 340 m). The site is a gently sloping ombrotrophic peat bog dominated by *Sphagnum* spp. mosses that experiences strong seasonal differences in temperature (mean monthly temperatures in January and July: -9.7 and 12.3°C, respectively, meteorological data 1999–2008, Abisko Scientific Research Station; a fuller site description is given in Dorrepaal et al. (2004)). The climate manipulation experiment was established in 2000 and consists of factorial combinations of summer treatments (ambient or warming), and winter/spring treatments (ambient and snow addition + spring warming), randomly assigned to hexagonal plots (2.5 m across) in five contiguous blocks parallel to the bog slope (i.e., 5 plots per 4 treatments = 20 plots total). Treatments are applied using open top chambers (OTCs) that increase average daily mean air temperature by 0.3–1.0°C in spring (April–June) and by 0.2–0.9°C in summer (June–October). These treatments have been shown to increase the rate of soil respiration and organic N cycling by 50–100% (Dorrepaal et al., 2009; Weedon et al., 2012). During winter, there is a passive accumulation of snow leading to an approximate doubling of the snow layer thickness and an increase of winter average soil temperature of 0.5–2.2°C (Dorrepaal et al., 2004, 2009).

Our sampling program was designed to characterize the soil bacterial community in plots subject to the four climate treatments described above. We targeted our sampling to the critical period of the winter–spring transition, when soils are thawing and there is turnover of microbial biomass and possible concomitant changes in bacterial community composition (Schmidt et al., 2007). Previous work has suggested that effects of warming on soil processes are related to dynamics in microbial populations in the early part of the growing season (Weedon et al., 2012). To characterize the bacterial community dynamics over this period, we sampled soil on three occasions in the spring–summer of 2011: April 19–22, May 2–4, and June 1–4. Over this period, the mean daily soil temperature rose from a uniform -0.1°C across the depth profile to 20 cm, to 4.8 and 1.3°C at 10 and 20 cm depth, respectively. On each sampling occasion, we took peat cores to a depth of 20 cm using a 2 cm diameter corer. To minimize the time between coring and nucleic acid extraction (see below), cores were taken four at a time and transported on ice back to the lab (500 m from the site) within 1 h for immediate extraction of nucleic acids.

### Nucleic Acid Extraction and cDNA Synthesis

Cores were hand-mixed, live moss and coarse roots removed, and four 0.3 g subsamples per core taken for nucleic acid extraction (total = 240 extractions) using the phenol–chloroform extraction protocol of Griffiths et al. (2000) with DEPC-treated reagents. Bead-beating was carried out using a table-top vortex and Lysing Matrix E tubes (QBiogene, Carlsbad, CA, USA). Precipitated nucleic acids were stored in 70% ethanol at -20°C and transported to Amsterdam, The Netherlands, for further analysis at the end of the field campaign (June 2011).

Prior to preparation for sequencing, the quantity and integrity of the extracted nucleic acids was checked by spectrophotometry (NanoDrop, Wilmington, DE, USA). Subsequently, the subsamples of each core were pooled and then split into two aliquots for either RNA- or DNA-based analyses. DNA was removed from the samples used for RNA analysis using an on-column DNase digestion with the RNeasy Protect Mini Kit (Qiagen, Valencia, CA, USA) following standard protocols. After verifying that no residual DNA remained (by negative PCR using universal bacterial primers (F357–R518; Muyzer et al., 1993), cDNA was synthesized with random hexamer primers using the RevertAid^TM^ Premium First Strand Synthesis Kit (Fermentas, Glen Burnie, MD, USA) following the manufacturer’s protocol.

### PCR and Illumina 16S rRNA Amplicon Sequencing

DNA and cDNA samples were prepared for Illumina sequencing following the protocol of Bartram et al. (2011). Briefly, each DNA/cDNA sample was amplified using custom primers that target the hypervariable V3 region of the bacterial 16S rRNA gene. These primers correspond to the primer pair F357 and R581, and include Illumina sequencing adapters and primer sequences, as well as one of 48 unique 6-bp barcode sequences. PCR was performed using GoTaq PCR Master mix (Promega, Madison, WI, USA), with ∼ 5 ng template (total DNA or cDNA), and the following thermocycler program: initial denaturation 95°C for 2 min; 25 cycles of 30 s at 95°C, 30 s at 50°C, and 45 s at 72°C; with a final elongation step of 5 min at 72°C. For convenience in further processing, PCR products were pooled into groups of six independent samples. Each of these pools was subsequently purified by gel extraction using the Qiagen Gel Extraction kit (Qiagen, Valencia, CA, USA). The molar concentration and purity of each of the clean amplicon pools was quantified on Bioanalyzer DNA chip (Agilent Technologies, Palo Alto, CA, USA) and the eight pools for each run were combined in equimolar ratios before being sequenced on the Illumina MiSeq platform (Illumina Technologies, San Diego, CA, USA), using 2 × 150 cycle paired-end settings. A total of 10,008,455 barcoded reads were obtained from three MiSeq runs that could be assigned to specific samples. There was a large degree of variation in sample coverage, ranging from 3.1 × 10^3^ to 2.14 × 10^5^ reads per sample (mean 7.0 × 10^4^, SD = 4.4 × 10^4^), although this large range was caused by a small number of outliers, and the central 90% of samples had a range of 2.2 × 10^4^ to 1.23 × 10^5^ reads per sample—with no systematic difference in read coverage due to sampling time, climate treatment, or RNA/DNA sample (see Supplementary Figure S1). To assess the reproducibility of the PCR and sequencing pipeline, several technical replicates (i.e., independent PCRs of the same nucleic acid sample using distinct barcode indices) were included in each run.

### Bioinformatics Workflow

Paired-end sequences were assembled using the USEARCH “merge” function (Edgar, 2013), with a maximum of one mismatch allowed in the overlapping region (85% of raw-reads retained). This was followed by quality filtering with the USEARCH “fastq_filter” function and maximum expected errors set at 0.05 (a stringent filter) which removed an additional 10% of the successfully merged reads. Operational taxonomic units (OTUs) were then defined over the complete sequence collection (RNA- and DNA-derived) using the UPARSE algorithm with 97% minimum similarity (Edgar, 2013), after removing all singleton reads. Chimeric sequences were removed with UCHIME (Edgar et al., 2011). A set containing representative sequences for each OTU was aligned using PyNAST (Caporaso et al., 2010a) using as a reference alignment the Green Genes (DeSantis et al., 2006) “core-set” as distributed with QIIME version 1.7.0 (Caporaso et al., 2010b). Sequences belonging to OTUs that failed to align with at least 75% sequence similarity, were most likely chimerical sequences or sequencing errors, and were removed from the dataset (499 OTUs representing 0.85% of successfully assembled reads). As PCR and sequencing errors can produce sequence errors leading to generation of spurious OTUs, we applied abundance filtering to the final dataset, removing all OTUs that were represented by less than 500 reads in the total dataset (see “Supplementary Presentation 1” for the choice of abundance threshold). This resulted in a dataset of 618 core OTUs (containing a total of 70% of the reads that successfully merged and passed quality filtering). Using the abundance filtered dataset, we generated a phylogenetic tree based on the aligned representative set using FastTree (Price et al., 2009), created separate OTU tables for DNA and RNA sequences, and assigned all OTUs to a taxonomic classification using the Ribosomal Database Project Bayesian classifier (Wang et al., 2007) with a threshold minimum confidence of 80%. Raw sequences are deposited in the NCBI Sequence Read Archive (accession number: SRP099222).

### Statistical Analyses

All analyses were performed in parallel on RNA- and DNA-based OTU tables. Due to the uneven coverage of sequence reads over the different barcoded samples, we performed all analyses on 100 randomly sub-sampled datasets, rarefied to an equal number of sequences per sample (7491 for DNA, 16,627 for RNA). This rarefaction depth was chosen to allow the inclusion of all but the two samples with lowest sequencing depth (see Supplementary Figure S1). Inclusion of these two samples would have strongly affected the coverage of the rarefaction. Phylogenetic dissimilarity matrices based on pairwise UniFrac distances between samples (Lozupone and Knight, 2005) were generated for each rarefied dataset. Cut-off filtering analyses showed that inferences based on unweighted Unifrac (i.e., ignoring read abundance) were extremely sensitive to the choice of abundance cut-off (see “Supplementary Presentation 1” in Supplementary Material). We therefore focused on the weighted version of the UniFrac metric, which is robust to the choice of cut-off. The effect of sampling time and climate change treatment on bacterial community composition was tested by permutational-MANOVA (PERMANOVA, Anderson, 2001) analysis of these UniFrac dissimilarity matrices using the ADONIS function in the vegan package of R (Oksanen et al., 2010; R Development Core Team, 2010), with block (i.e., spatial location at the experimental site), climate change treatment, sampling date, and date × treatment interaction as fixed factors. We report the mean *R*^2^ and permutation *P*-values from analyses conducted separately on each of the 100 rarefied subsets. Relationships between samples from different times or climate change treatments were visualized by Principal Coordinates Analysis (PCoA) ordinations of the UniFrac dissimilarity matrices. Differences in multivariate dispersion between levels of treatment factors were evaluated using the permutational analyses of multivariate dispersion (PERMDISP, Anderson, 2006) implemented in the vegan R package (Oksanen et al., 2010) Reproducibility of the PCR and sequencing pipeline was confirmed by the small standard errors of technical replicates in the appropriate PCoA plot (see Supplementary Figure S2).

Community composition variation was more closely associated with sampling date than treatment effects (see Results). To better characterize this pattern, we analyzed each OTU separately to identify those contributing to the seasonal shift in community composition. Our goal was to classify each OTU as “increasing,” “decreasing,” or “neutral” with respect to sampling time. For each of the 100 rarefied datasets (for both RNA- and DNA-based analyses), we calculated the slope and *P*-value of linear regression of log-transformed abundance as a function of sampling time for each OTU. We then applied the Benjamini–Hochberg (Benjamini and Hochberg, 1995) false discovery rate (FDR) correction to the resulting *P*-values (each averaged over the 100 rarefaction datasets). OTUs with a significantly (after FDR correction) positive or negative slopes were classified as “increasing” or “decreasing,” respectively.

## Results

### Phylum-Level Taxonomic Composition of Samples

**FIGURE 1:**
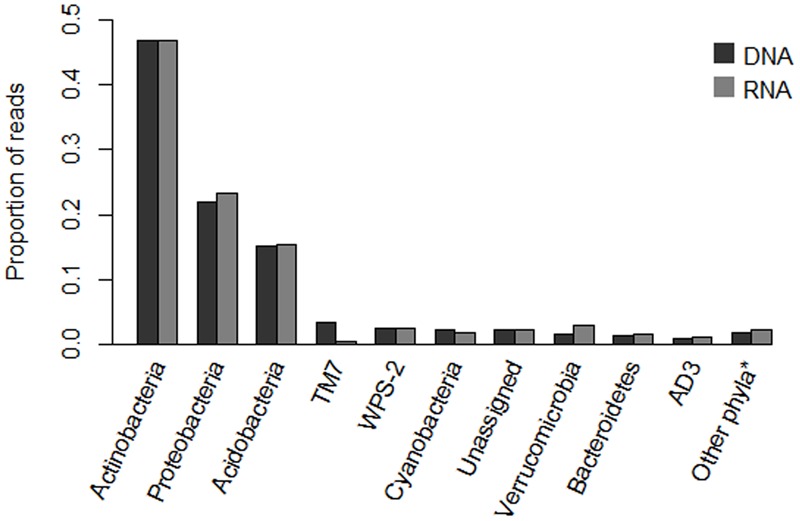
**Assignment of amplicon sequencing reads in the core 618 OTU dataset to phyla level classification, for DNA- and RNA-based analyses, respectively.** Category “Other^∗^” contains phyla representing less than 1% of sequences, and contains members of the phyla Planctomycetes, Armatimonadetes, Firmicutes, Elusimicrobia, Chlamydiae, Chlorobi, Chloroflexi and candidate divisions TM6 and SC3.

Phyla-level assignments of sequences in the core 618 OTU dataset were broadly similar between the DNA- and RNA-based analyses (**Figure 1**). The majority of sequences were assigned to the phyla Actinobacteria (46.9 and 46.7% of DNA and RNA sequences, respectively), Proteobacteria (21.9 and 23.2%), and Acidobacteria (15.2 and 15.3%). Within the phylum Proteobacteria, members of the class Alphaproteobacteria dominated the sequence counts (77.8% of all Proteobacteria in DNA, 64.3% in RNA), followed by Deltaproteobacteria, which was strongly overrepresented in the RNA sequences (3.7% versus 23.0%), with the remainder assignable to Gammaproteobacteria (15.4 and 10.6%), Betaproteobacteria (2.5 and 1.8%) or not assignable below phylum level (0.5 and 0.2%). The phyla Cyanobacteria, Verrucomicrobia, Bacteroidetes and candidate divisions WPS-2 and TM7 each contributed between 1 and 4% of sequences in both data sets (with the exception of a relative underrepresentation of candidate division TM7 and Cyanobacteria in RNA relative to DNA datasets). Of the remaining circa 5% of sequences, 2.3% (both DNA and RNA) could not be assigned to phyla level at the 80% confidence level, while other sequences were distributed over the phyla Planctomycetes, Armatimonadetes, Firmicutes, Elusimicrobia, Chlamydiae, Chlorobi, Chloroflexi and candidate divisions AD3, TM6, and SC3, with each phylum contributing less than 0.9% of the total sequences.

### Effects of Climate Change Treatments and Sampling Time on Bacterial Community Composition

**FIGURE 2:**
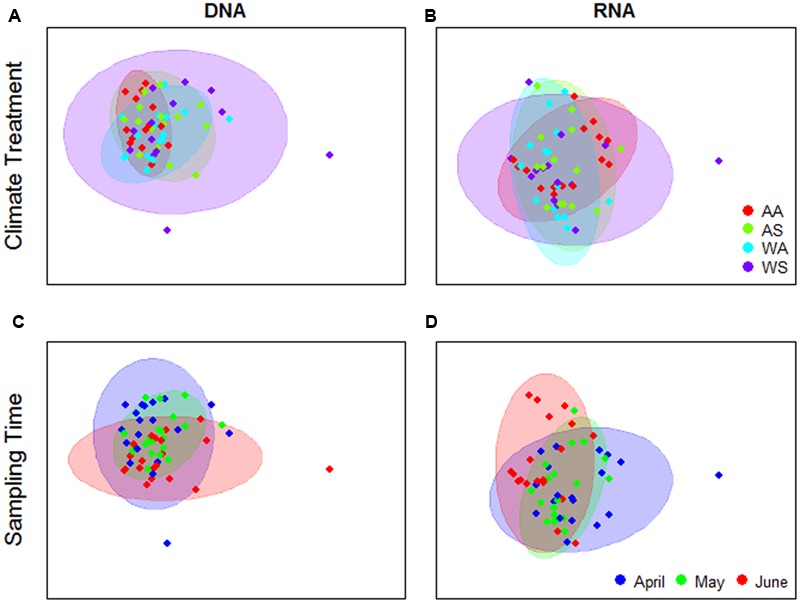
**Principal Coordinates Analysis (PCoA) ordinations of UniFrac distance matrices generated from RNA or DNA bacterial 16S V3 amplicon sequences.** Ordinations were performed on the mean of 100 matrices per sample type, corresponding to rarefactions to ensure even sample coverage. Colors of points and 90% confidence ellipses correspond to climate change treatment **(A,B)** or sampling time **(C,D)**. Treatment codes—first letter: A, summer ambient; W, summer warming; second letter: A, spring/winter ambient; S, spring warming, winter snow accumulation.

Analysis of UniFrac distance matrices by PERMANOVA indicated some evidence for an effect of the climate change treatments on the bacterial community composition in the DNA-based analyses, but not in the rRNA-based analyses (**Table 1**). However, in the former case, the pattern does not appear to be driven by a consistent shift in community composition due to the treatments (location in ordination space), but rather through treatment effects on community composition variability (i.e., as indicated by dispersion in ordination space) (**Figure 2A**). This is confirmed by the results of the PERMDISP test which showed significant differences in group dispersions when classified by treatment for DNA data only (PERMDISP *F*_3,55_ = 4.37, *P* = 0.008) No differences in dispersion were found in the RNA data (PERMDISP *F*_3,56_ = 1.39, *P* = 0.25). The interaction between treatment and time was not significant in any of the PERMANOVA analyses (mean permutation *P* > 0.05, **Table 1**), implying that there was no evidence for climate treatment-related temporal shifts in bacterial community composition (**Figures 2A,B**).

**Table 1:** Table of *R*^2^ values (as %) for PERMANOVA model factors based on abundance weighted UniFrac dissimilarity matrices.

Source of variation	DNA	RNA
Space (block)	14.2^∗^	12.1^∗^
Time (month)	7.5^∗^	10.1^∗^
Treatment	8.3^∗^	4.8
Time × treatment	6.6	7.2
Residual variation	63.4	65.8

*^∗^*P* < 0.01.*

For both DNA- and rRNA-based analyses, space (expressed as treatment block) and sampling time explained a statistically significant fraction of the dissimilarity matrix structure (**Table 1**). Sampling time effects were most pronounced in RNA compared to DNA (**Figures 2C,D**). For RNA, there is a visible shift in the central location of the samples in ordination space with time (**Figure 2D**, but this shift is subtle, reflected in the high residual variation (∼65%) and the lack of discrete clusters in the ordination. PERMDISP analyses showed no significant differences in group dispersion when classified by sampling time, or treatment block (not shown).

### Selectively Responding OTUs

**FIGURE 3:**
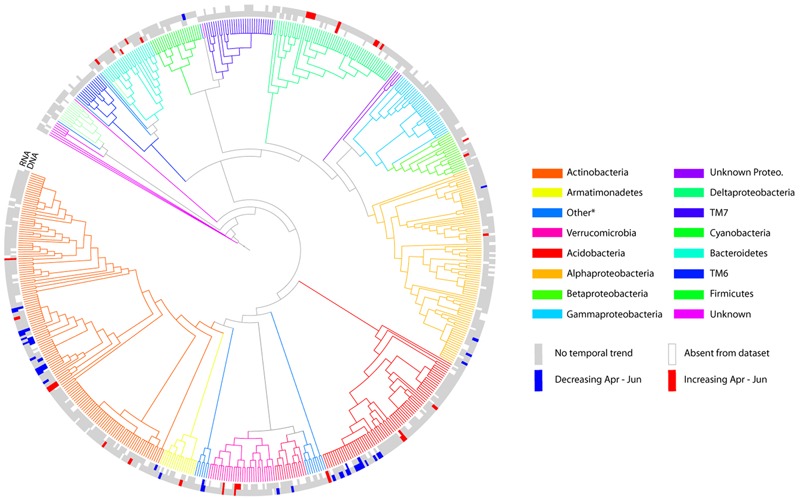
**Tree representation of core 618 OTUs (represented by >500 reads in DNA and/or RNA subsets), based on alignment of V3 region of the 16S rRNA gene.** The colour of branches indicates phylum level assignment by tree-based classification using RaXML, pplacer and the Green Genes 85% OTU tree. Branch-lengths are homogenized for legibility, and are therefore not proportional to phylogenetic distance used to calculate UniFrac metrics for beta-diversity analyses. Tips are labeled blue for OTUs that decreased over the sampling period, red for increasers, and gray for no significant temporal pattern. The inner ring shows results for DNA analyses, the outer ring RNA analyses. More detailed taxonomic assignment and relative abundances of significant indicator OTUs are given in Supplementary Table S1.

Given that sampling time explained a higher proportion of variance in community composition relative to climate-change treatments, we focused on this temporal effect by determining which OTUs show shifts in relative abundance across the sampling period (**Figure 3**). For DNA-based analyses, a total of 26 OTUs out of 618 OTUs showed significant variation (after FDR correction) in relative abundance from April to June, of which 14 increased over time, and 12 decreased. The number of significantly changing OTUs was greater for the RNA-based analysis: 25 significantly increased in relative abundance through time and 34 decreased. There was some overlap between the two analyses, eight of the increasing OTUs and eight of the decreasing OTUs were identified by both the DNA- and RNA-based analyses. There was also some degree of phylogenetic coherence in these temporal patterns: for example, the groups Bacteroidetes, Verrucomicrobia, and Deltaproteobacteria contained only increasing OTUs in both DNA and/or RNA datasets (taxonomic assignments for indicator species are in Supplementary Table S1). In other phylogenetic groups such as Actinobacteria, Acidobacteria, and Alphaproteobacteria both increasing and decreasing taxa were identified. However, the vast majority of OTUs showed no significant pattern of variation related to sampling time, indicating a relatively stable bacterial community composition. In contrast to the temporal changes detected, there were no OTUs identified that showed a significant response to climate treatment.

## Discussion

Despite previous observations of consistently strong effects (50–100% increases) of the experimental treatments on C and N cycles in our system, we did not detect strong effects of the same treatments on the composition of the bacterial community (**Table 1** and **Figure 2**). Although treatment effects on phylogenetic community structure were found to be statistically significant for DNA-based analyses (**Table 1**), these appear to be related to treatment effects on variance, rather than differences in the mean composition of the communities (**Figure 2**; see also Anderson, 2001). Moreover, the treatment effect was consistently weaker than spatial (over 10s of meters) and temporal (over 3 months) variation (**Table 1**). Some OTUs showed significant temporal trends in relative abundance (**Figure 3**), and, interestingly, these OTUs often showed some degree of phylogenetic clustering (cf. Amend et al., 2016). Nevertheless, bacterial community structure was in general unresponsive to both experimentally applied climate change, as well as seasonal variation in environmental conditions.

Our failure to detect a clear directional response of the bacterial community related to climate change treatment is not a result of low-statistical power. To illustrate, while we observed an average within-treatment weighted Unifrac distance of 0.09, other studies with 16S amplicon community profiles from soil environments have reported within-site mean distances as high as 0.4–0.6 (Ferrenberg et al., 2013). Moreover, our analyses based on 16S rRNA led to qualitatively very similar conclusions as those based on 16S rRNA genes (DNA). Relative stability of bacterial community composition could be a consequence of high-levels of bacterial dormancy, and/or long-term preservation of DNA from non-living microorganisms (Jones and Lennon, 2010). However, the congruent results from both DNA and RNA analyses, and the long-term nature of the experimental manipulations (>10 years) support our interpretation that the bacterial community structure is either non-responsive to the experimental climate change—or if it was once sensitive, has subsequently returned to the undisturbed state (i.e., resilient; Allison and Martiny, 2008) while consistently amplifying biogeochemical fluxes.

Several recent studies have reported significant shifts in microbial community composition and/or functional gene abundance in response to experimental warming (Yergeau et al., 2012; Luo et al., 2014; Xiong et al., 2014), although in most cases the detected effect sizes were rather modest (e.g., a mean 2% increase in phyla-level abundances; Luo et al., 2014) and further analysis is required to elucidate how this climate-related variation compares to underlying spatial and temporal variation. On the other hand, the lack of detectable effect of experimental warming on bacterial community composition agrees with results from previous studies of soil microbes in grasslands (Penton et al., 2013), and temperate upland soils (Kuffner et al., 2012). In a somewhat different context, Cruz-Martinez et al. (2009) also found community resistance to precipitation manipulation—despite considerable treatment effects on overlying vegetation. These results imply that the sensitivity of bacterial communities to warming (and other disturbances) is likely to be generally small, but context dependent.

The lack of effects on bacterial community composition, in a system where climate treatments had previously been shown to strongly affect soil processes (Dorrepaal et al., 2009; Weedon et al., 2012), also raises questions about the decoupling of functional and compositional stabilities (Yergeau et al., 2012). In a meta-analysis, Shade et al. (2012) reported that out of 378 disturbance studies examining microbial communities, 56% showed a response at the level of microbial community function (e.g., respiration, enzyme activities), but only just over half of these functional effects were accompanied by detectable shifts in microbial community composition. Similarly, a recent meta-analysis of experimental studies showed that a significant link between measures of community structure and community functions was found in only a minority of studies (Bier et al., 2015). In the light of these findings and the results presented in the current study, we suggest that (as in the case of community resistance and resilience; De Vries and Shade, 2013) it may be helpful to shift the focus of investigations from asking “are changes in microbial function associated with changes in microbial community composition?” to “*in which contexts* are changes in microbial function associated with changes in microbial community composition?” (cf. Krause et al., 2014).

**FIGURE 4:**
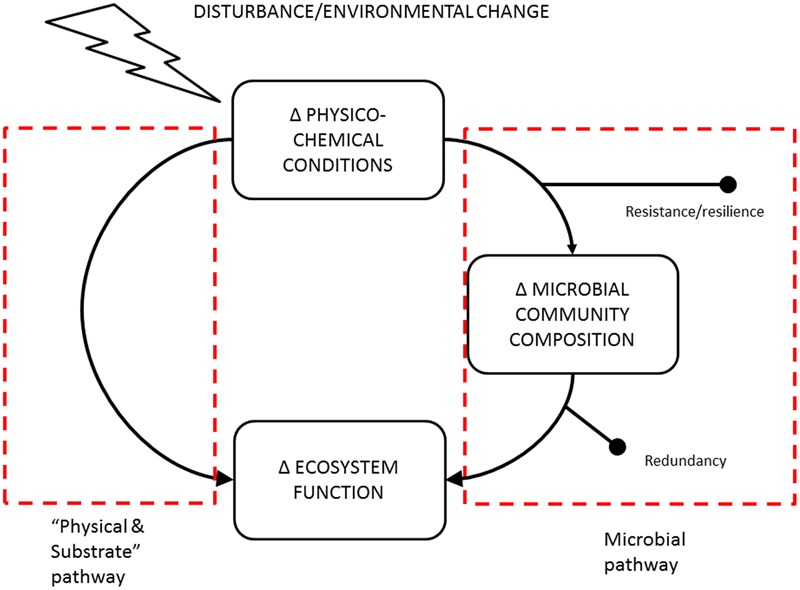
**Schematic describing the two major pathways by which disturbance or change in environmental conditions can alter microbe-mediated ecosystem processes.** The right side of is adapted from Allison and Martiny (2008).

A commonly used framework for relating environmental change to microbial community composition and associated functions was proposed by Allison and Martiny (2008) (see right side of **Figure 4**). Microbial community composition will mediate ecosystem process responses to environmental change as long as the communities are not resistant, resilient or functionally redundant. This “Microbial Pathway” has received primary attention in recent studies of the relationship between disturbance and changes to microbe-mediated processes (Allison and Martiny, 2008; Shade et al., 2012; De Vries and Shade, 2013). For the purposes of interpreting our results, we consider an alternative “Physical and Substrate” alternative pathway linking environmental change and ecosystem functions. That is, that changes in physico-chemical conditions and substrate supply rates can also influence the rate of an ecosystem process directly without changes to the microbial community composition (left side of **Figure 4**). The question of interpreting the mixed evidence relating microbial community composition to ecosystem processes (Bier et al., 2015; Graham et al., 2016) can then be framed in terms of the relative importance of these two pathways. We propose that for C and N cycling under simulated climate change in the peatland system we examined, the “Physical and Substrate” pathway is more important than community composition effects. We suggest two possible explanations for this.

Firstly, the ecosystem processes in question—cycling of organic N and C—are most likely carried out by a broad range of microorganisms, with a correspondingly broad range of environmental tolerances (Prosser, 2012; Schimel and Schaeffer, 2012). This leads to a weak coupling between changes to physical conditions and the aggregate functional potential of the microbial community, and therefore no clear relationship between community composition and measured functions. Schimel and Schaeffer (2012) have proposed that many component processes of the soil carbon cycle can be classified as phylogenetically “broad.” This can lead to situations such as reported by Tveit et al. (2013) who found that at the level of the metatranscriptome related to C cycling, soil communities from high-arctic tundra permafrost soils are indistinguishable from those in temperate grasslands. In such situations, magnitudes of ecosystem processes (e.g., soil respiration, decomposition rates) may be more determined by activity and/or growth responses of the bacterial community to physical parameters than by the composition of the microbial community. Conversely, rates of “narrow” functions, performed by a relatively smaller proportion of the microbial community, such as acetoclastic methanogenesis (Godin et al., 2012), ammonia oxidation (Kowalchuk and Stephen, 2001), and methane oxidation (Bodelier et al., 2012), may be more strongly coupled to community composition. In the context of the current study, this distinction between “broad” and “narrow” functions could be tested by examining the relationship between climate treatment and the composition and activity of microorganisms involved in more phylogenetically limited functions such as methane production and consumption, or nitrous oxide emissions.

Secondly, our data support the conclusion that the bacterial community in our peatland is resistant or resilient with regard to the disturbance introduced by the climate treatments. This is commonly observed under experimental climate change experiments with small temperature increases (Kuffner et al., 2012; Penton et al., 2013). If the microbial community is resistant to disturbance (or, if sensitive, shows subsequent resilience), then it follows that any consequences of that disturbance for ecosystem processes cannot be explained by changes in community composition. In contrast, for highly sensitive communities, loss or suppression of particular functional groups (and therefore shifts in community structure) in response to disturbance can have consequences for ecosystem processes. For example, it has been proposed that suppression of saprophytic fungal communities by high nutrients and low pH may explain observed reductions in forest soil respiration in response to nitrogen deposition (Janssens et al., 2010). In this context, it should be fruitful to explore which characteristics of environments or communities determine resistance or sensitivity, e.g., relative proportions of oligotrophic versus copiotrophic taxa (De Vries and Shade, 2013), soil chemical properties (Griffiths and Philippot, 2013), or food web structure (Neutel et al., 2002). For the *Sphagnum* peatlands sampled in the current study, it could be argued that a fairly strong environmental filter imposed by the low pH and high concentration of secondary compounds derived from the *Sphagnum* peat have already selected for a resistant microbial community (Opelt et al., 2007).

We conclude that the combination of a phylogenetically broad ecosystem process and a resistant bacterial community results in a system where bacterial community composition is not an important mediator of the effects of climate warming on peatland C and N cycling. Indeed, we see the lack of climate sensitivity of bacterial community composition as evidence that warming effects on substrate supply can better explain the climate sensitivity of C and N cycling rates (Weedon et al., 2013, 2014). We suggest that our distinction (**Figure 4**) between systems where composition is an important mediator, from those where physical and substrate supply plays a more important role, may be a useful conceptual framework for interpreting past and future datasets (Graham et al., 2014, 2016; Bier et al., 2015). Important to note is that even when the physical and substrate pathway dominates, this does not imply that microbes are unimportant. Rather the size and/or aggregate activity of microbial populations (driven by substrate supply) may be a more important predictor of ecosystem processes in these contexts (Serna-Chavez et al., 2013).

Several caveats and potential alternative interpretations should be considered in relation to our conclusions. Firstly, our data set cannot exclude the possibility that warming effects on soil C and N cycling was mediated by microorganisms not detected with our profiling method, in particular fungi and other eukaryotes. Although fungi are known to be present and active in ombrotrophic mires (Thormann, 2006), there is evidence that microbial activity in boreal peatlands is dominated by bacteria (Myers et al., 2012). Further research into the relative stability of bacterial versus eukaryotic soil microorganisms, and the accompanying functional consequences, would help to further illuminate this issue.

Secondly, the large amount of unexplained variation in bacterial community composition (**Table 1**) could imply that warming effects are obscured by variation driven by small scale differences in other environmental drivers, such as soil pH or vegetation. Previous studies in the same system have shown that soil pH is fairly uniform across the site, and not affected by climate treatment (Lang et al., 2009). Plant community composition is similarly not affected by climate treatment (Keuper et al., 2011), but it could be expected that plants influence bacterial communities at small scales, by litter and rhizosphere effects (Urbanová et al., 2015), independent of treatments, thus potentially obscuring any treatment effect. This can only be analyzed with plant community data matched to our microbial samples. However, even if this were the case, it would only further underline the absence of strong effects of the climate treatment on bacterial communities.

Lastly, our dataset does not allow a direct comparison between process measurements and bacterial communities, due to a temporal mismatch in sampling. Carbon cycle effects were measured in the period 2004–2008 (Dorrepaal et al., 2009), N cycle effects in the summer of 2009 (Weedon et al., 2012), and sampling for the bacterial community profiles conducted in 2011 (present study). In inferring a lack of link between process rates and bacterial communities, we are therefore making the assumption that the climate effects on process rates have persisted over the 2–3 years separating the community and process measurement. Although we lack the data to directly test this assumption, for our main conclusions to change it would be necessary for both bacterial communities to covary with process rate responses to treatments in the period 2004–2009, and for composition and process responses to cease after 2009. A lack of treatment effects on community profiles in samples from 2009 based on phyla-specific qPCR (admittedly, a much coarser technique than amplicon sequencing) makes the former seem unlikely (Weedon et al., 2012); and the detection of ongoing treatment effects on aspects of ecosystem C cycling extending to 2011 (Hicks Pries et al., 2015) could be taken as partial evidence against the latter.

As more studies seek to link microbial community composition to ecosystem processes, we suggest that framing the questions in terms of the relative importance of community composition effects versus physical and substrate effects may provide some general insights. The novelty and data richness of sequencing and other –omics approaches should not obscure the fact that in some environments, and for some ecosystem processes, simpler models, without community effects, may be sufficient for understanding controls of process rates. Identifying the contexts where compositional effects are more or less important will be an important step in building a predictive soil microbial ecology.

## Author Contributions

GK, RA, JW, and PvB designed the experiment. SF and JW performed the field and lab work. JW and PvB performed the bioinformatic and statistical analyses. JW, PvB, GK, RA, and WR interpreted the data. JW and PvB wrote the first draft of the manuscript, and all authors contributed substantially to revisions.

## Conflict of Interest Statement

The authors declare that the research was conducted in the absence of any commercial or financial relationships that could be construed as a potential conflict of interest.
