# Supplementary material for: Compositional Stability of the Bacterial Community in a Climate-Sensitive Sub-Arctic Peatland
Source: Front Microbiol. 2017 Mar 7;8:317. doi: 10.3389/fmicb.2017.00317 (PMC5339224; doi:10.3389/fmicb.2017.00317)
Supplement: Supplementary file 1 [file Table_1.DOCX]

**Table S1 Taxonomic assignments of ‘indicator’ OTUs**

The table below shows taxonomic assignments for the 69 OTUs identified as “indicators” – that is that showed statistically significant (after false discovery rate correction) increase or decrease in relative abundance over the three sampling periods in the experiment. Assignments were made using the RDP classifier, with a 80% confidence threshold for assignment to a given taxonomic level. For each OTU the fold change in mean abundance over the sampling period (defined as A/B, where A is the highest of April or June mean relative abundance, and B is the other value, and negative fold changes indicate reductions over the sampling period so 2 = a doubling in mean relative over sampling period, and -1.5 = a 50% reduction). Overall relative abundances (as % of the corresponding DNA or RNA rarefied OTU tables) are also given.

| **OTU Identifier** | **Phylum** | **Class** | **Order** | **Family** | **Genus** | **Fold change** | | **Relative abundance (%)** | |
| --- | --- | --- | --- | --- | --- | --- | --- | --- | --- |
| **DECREASING WITH TIME (RNA & DNA)** | | | | | | *RNA* | *DNA* | *RNA* | *DNA* |
| OTU_5107 | Acidobacteria | Acidobacteriia | Acidobacteriales | Acidobacteriaceae |  | -1.6 | -1.4 | 1.01 | 1.04 |
| OTU_7 | Acidobacteria | Acidobacteriia | Acidobacteriales | Acidobacteriaceae |  | -1.7 | -1.4 | 1.53 | 1.29 |
| OTU_104 | Actinobacteria | Acidimicrobiia | Acidimicrobiales |  |  | -1.4 | -2.3 | 0.1 | 0.08 |
| OTU_1637 | Actinobacteria | Acidimicrobiia | Acidimicrobiales |  |  | -1.8 | -1.9 | 0.21 | 0.11 |
| OTU_2 | Actinobacteria | Acidimicrobiia | Acidimicrobiales |  |  | -1.3 | -1.4 | 5.45 | 4.72 |
| OTU_7337 | Actinobacteria | Acidimicrobiia | Acidimicrobiales |  |  | -1.7 | -1.5 | 0.73 | 0.36 |
| OTU_6468 | Actinobacteria | Acidimicrobiia | Acidimicrobiales |  |  | -1.5 | -1.8 | 1.12 | 0.78 |
| OTU_102 | Planctomycetes | Planctomycetia | Gemmatales | Isosphaeraceae |  | -1.9 | -2.4 | 0.12 | 0.06 |
| **DECREASING WITH TIME DNA ONLY** | | | | | |  | |  | |
| OTU_7674 | Actinobacteria | Acidimicrobiia | Acidimicrobiales |  |  | -1.8 | | 0.18 | |
| OTU_6972 | Actinobacteria | Acidimicrobiia | Acidimicrobiales |  |  | -1.6 | | 0.68 | |
| OTU_7146 | Actinobacteria | Acidimicrobiia | Acidimicrobiales |  |  | -1.7 | | 0.16 | |
| OTU_25 | Actinobacteria | Actinobacteria_1 |  |  |  | -1.5 | | 0.62 | |
| **DECREASING WITH TIME RNA ONLY** | | | | | |  | |  | |
| OTU_170 | Cyanobacteria | 4C0d-2 | MLE1-12 |  |  | -2.5 | | 0.05 | |
| OTU_3785 | Proteobacteria | Alphaproteobacteria | Rhodospirillales_1 | Acetobacteraceae |  | -1.2 | | 0.76 | |
| OTU_6313 | Proteobacteria | Alphaproteobacteria | Rhodospirillales_1 | Acetobacteraceae |  | -2.4 | | 0.04 | |
| OTU_7284 | Proteobacteria | Alphaproteobacteria | Rhodospirillales_1 | Acetobacteraceae |  | -1.3 | | 1.08 | |
| OTU_7664 | Proteobacteria | Alphaproteobacteria | Caulobacterales | Caulobacteraceae |  | -1.8 | | 0.3 | |
| OTU_7554 | Acidobacteria | Acidobacteriia | Acidobacteriales | Acidobacteriaceae |  | -2.2 | | 0.09 | |
| OTU_7658 | Acidobacteria | Acidobacteriia | Acidobacteriales | Acidobacteriaceae |  | -1.7 | | 0.37 | |
| OTU_6492 | Acidobacteria | Acidobacteriia | Acidobacteriales | Acidobacteriaceae |  | -1.9 | | 0.06 | |
| OTU_5446 | Acidobacteria | Acidobacteriia | Acidobacteriales | Acidobacteriaceae |  | -1.6 | | 0.87 | |
| OTU_353 | Acidobacteria | Acidobacteriia | Acidobacteriales | Acidobacteriaceae |  | -1.7 | | 0.07 | |
| OTU_26 | Acidobacteria | Acidobacteriia | Acidobacteriales | Acidobacteriaceae |  | -1.5 | | 0.59 | |
| OTU_6979 | Acidobacteria | Acidobacteriia | Acidobacteriales | Acidobacteriaceae |  | -1.6 | | 0.51 | |
| OTU_6143 | Acidobacteria | Acidobacteriia | Acidobacteriales | Acidobacteriaceae |  | -1.8 | | 0.24 | |
| OTU_6792 | Acidobacteria | Acidobacteriia | Acidobacteriales | Acidobacteriaceae |  | -1.7 | | 0.34 | |
| OTU_4178 | Acidobacteria | Acidobacteriia | Acidobacteriales | Acidobacteriaceae |  | -1.5 | | 0.3 | |
| OTU_3254 | Acidobacteria | Acidobacteriia | Acidobacteriales | Acidobacteriaceae |  | -1.8 | | 0.08 | |
| OTU_4385 | Actinobacteria | Acidimicrobiia | Acidimicrobiales |  |  | -1.3 | | 0.23 | |
| OTU_4586 | Actinobacteria | Acidimicrobiia | Acidimicrobiales |  |  | -2.3 | | 0.25 | |
| OTU_7584 | Actinobacteria | Acidimicrobiia | Acidimicrobiales |  |  | -2.1 | | 0.03 | |
| OTU_14 | Actinobacteria | Acidimicrobiia | Acidimicrobiales |  |  | -1.3 | | 1.23 | |
| OTU_33 | Actinobacteria | Acidimicrobiia | Acidimicrobiales |  |  | -1.7 | | 0.43 | |
| OTU_7909 | Actinobacteria | Acidimicrobiia | Acidimicrobiales |  |  | -1.7 | | 0.04 | |
| OTU_70 | Armatimonadetes | [Fimbriimonadia] | [Fimbriimonadales] | [Fimbriimonadaceae] | Fimbriimonas | -1.7 | | 0.18 | |
| OTU_507 | Planctomycetes | Planctomycetia | Gemmatales | Isosphaeraceae |  | -1.8 | | 0.13 | |
| OTU_16 | WPS-2 |  |  |  |  | -1.4 | | 1.25 | |
| OTU_3675 | WPS-2 |  |  |  |  | -1.6 | | 0.33 | |

| **OTU Identifier** | **Phylum** | **Class** | **Order** | **Family** | **Genus** | **Fold change** | | **Relative abundance (%)** | |
| --- | --- | --- | --- | --- | --- | --- | --- | --- | --- |
| **INCREASING WITH TIME (RNA & DNA)** | | | | | | *RNA* | *DNA* | *RNA* | *DNA* |
| OTU_127 | Proteobacteria | Deltaproteobacteria | Myxococcales |  |  | 10.6 | 11.8 | 0.18 | 0.04 |
| OTU_2841 | Acidobacteria | Acidobacteriia | Acidobacteriales |  |  | 18.7 | 13.9 | 0.03 | 0.06 |
| OTU_94 | Elusimicrobia | Elusimicrobia | Elusimicrobiales |  |  | 1.8 | 2.8 | 0.08 | 0.08 |
| OTU_213 | Actinobacteria | Acidimicrobiia | Acidimicrobiales | EB1017 |  | 12.4 | 13.4 | 0.03 | 0.06 |
| OTU_1101 | Actinobacteria | Acidimicrobiia | Acidimicrobiales | EB1017 |  | 16.3 | 29.5 | 0.02 | 0.05 |
| OTU_191 | Actinobacteria | Actinobacteria | Actinomycetales_1 | Propionibacteriaceae |  | 5 | 4.2 | 0.03 | 0.04 |
| OTU_87 | Verrucomicrobia | Opitutae |  |  |  | 2.1 | 2.2 | 0.18 | 0.27 |
| OTU_119 | Verrucomicrobia | [Pedosphaerae] | [Pedosphaerales] |  |  | 4.5 | 4.7 | 0.15 | 0.19 |
| **INCREASING WITH TIME DNA ONLY** | | | | | |  | |  | |
| OTU_445 | Bacteroidetes | Cytophagia | Cytophagales | Cytophagaceae |  | 67.5 | | 0.02 | |
| OTU_4055 | Bacteroidetes | [Saprospirae] | [Saprospirales] | Chitinophagaceae |  | 3.2 | | 0.02 | |
| OTU_69 | Proteobacteria | Betaproteobacteria | Burkholderiales |  |  | 1.9 | | 0.17 | |
| OTU_4894 | Proteobacteria | Alphaproteobacteria | Rhizobiales | Hyphomicrobiaceae_1 | Rhodoplanes | 15 | | 0.06 | |
| OTU_368 | Verrucomicrobia | Opitutae |  |  |  | 6.4 | | 0.04 | |
| OTU_159 | Verrucomicrobia | Opitutae |  |  |  | 2 | | 0.1 | |
| **INCREASING WITH TIME RNA ONLY** | | | | | |  | |  | |
| OTU_412 | Bacteroidetes | [Saprospirae] | [Saprospirales] |  |  | 2 | | 0.02 | |
| OTU_1080 | Bacteroidetes | Sphingobacteriia | Sphingobacteriales | Sphingobacteriaceae |  | 2.8 | | 0.02 | |
| OTU_283 | Bacteroidetes | Sphingobacteriia | Sphingobacteriales |  |  | 2.3 | | 0.02 | |
| OTU_292 | Proteobacteria | Betaproteobacteria | Gallionellales | Gallionellaceae | Gallionella | 2 | | 0.06 | |
| OTU_395 | Proteobacteria | Deltaproteobacteria | Myxococcales |  |  |  | | 0.04 | |
| OTU_4390 | Proteobacteria | Deltaproteobacteria | Myxococcales |  |  | 3.4 | | 0.13 | |
| OTU_5780 | Proteobacteria | Deltaproteobacteria | Myxococcales |  |  | 2.4 | | 0.07 | |
| OTU_6661 | Proteobacteria | Deltaproteobacteria | Myxococcales |  |  | 37.8 | | 0.03 | |
| OTU_409 | Proteobacteria | Deltaproteobacteria | Myxococcales |  |  | 19.4 | | 0.03 | |
| OTU_95 | Proteobacteria | Deltaproteobacteria | Myxococcales |  |  | 3.1 | | 0.18 | |
| OTU_461 | Proteobacteria | Deltaproteobacteria | Myxococcales |  |  | 14.9 | | 0.03 | |
| OTU_306 | Acidobacteria | Acidobacteriia | Acidobacteriales |  |  | 9 | | 0.07 | |
| OTU_176 | Acidobacteria | Solibacteres |  |  |  | 2.6 | | 0.11 | |
| OTU_13 | Actinobacteria | Acidimicrobiia | Acidimicrobiales |  |  | 1.5 | | 0.43 | |
| OTU_393 | Actinobacteria | Thermoleophilia | Solirubrobacterales |  |  | 3.1 | | 0.08 | |
| OTU_125 | Actinobacteria | Thermoleophilia | Gaiellales | Gaiellaceae |  | 5.5 | | 0.02 | |
| OTU_1 | Armatimonadetes | 0319-6E2 |  |  |  | 1.3 | | 10.69 | |
