# Supplementary material for: Compositional Stability of the Bacterial Community in a Climate-Sensitive Sub-Arctic Peatland
Source: Front Microbiol. 2017 Mar 7;8:317. doi: 10.3389/fmicb.2017.00317 (PMC5339224; doi:10.3389/fmicb.2017.00317)
Supplement: Supplementary file 2 [file Presentation_1.pdf]

Appendix S1 *Analyses of the effects of varying the abundance cutoff on subsequent inference*

Introduction

An important problem in the bioinformatic and statistical analysis of microbial community data generated by next generation sequencing techniques is the correct procedure for dealing with low-abundance OTUs in statistical analyses. The rank-abundance curve of amplicon-based OTUs generally has an extremely long tail (e.g. > 50% of unique sequences represented by a single amplicon read). Developing objective means for distinguishing how much of this long tail reflects biological reality (the "rare biosphere"), or is a result of methodological artifacts, remains an active area of research. This is because generating an amplicon based community profile necessarily involves a number of "sampling" processes (taking a sample from the environment, but also DNA from sample, amplicon from DNA, read from amplicon), each with potentially significant biases, and most associated with a degree of error. The method of processing sequences and defining OTUs can also have large effects on the composition and relative abundance of different OTUs, and therefore on some of the metrics used for cross-sample comparison and inference.

One of the proposed practices for dealing with the long tail, is to discard all OTUs from which the observed number of reads falls below a pre-specified cutoff. The cutoff can be set on a per-experiment basis through the use of "mock" communities of known composition. The cutoff that is appropriate for recovering the known diversity of the mock community can then be applied to the biological samples. As well as having its own drawbacks, this method is not applicable *post hoc* in cases where a mock community was not included in the PCR and sequencing workflow. In such cases investigators often apply an arbitrary with little or no justification. We agree this that is often unavoidable, but we also think that it is important to examine the potential consequences of this arbitrary choice.

For the purposes of the current study, we have performed a sensitivity analysis to determine to what extent our statistical inferences are affected by the choice of abundance cutoff. Briefly, we repeated the statistical analyses described in the main text, using a range of abundance cutoffs and compared the results in terms of the effect on the associated test statistics, as well as on their reproducibility (under repeated rarefactions of the data). Here we will briefly discuss the methodology and present the main results.

Methods

Data used are identical to those used in the main text, we performed parallel analyses on the DNA and RNA-based subsets. We wrote a small collection of R and Python scripts to automate the sensitivity analyses. Scripts are available from the authors on request, here we summarize the workflow as pseudocode:

INPUTS:

cutofffile.txt File containing list of (numeric) cutoffs – a number *n* such that only OTUs with a total number of reads > *n* will be retained.

master.biom master OTU table – containing raw read abundances for each sample x OTU combination

treefile.tre master tree file, a phylogenetic tree containing all the OTUs in *master.biom*

OUTPUT:

outputN/ output directory containing all output files for cutoff level *N*

WORKFLOW:

For each **cutoff** in ***cutofffile.txt***

1. Create directory for output
2. Filter ***master.biom*** at abundance level **cutoff -> filtered_table.biom**
3. Convert **filtered_table.biom** to normal delimited text OTU table -> **filtered_table.otu**
4. Check appropriate rarefaction depth with an R script (returns minimum column sum from **filtered_table.otu**)
5. Rarefy **filtered_table.biom** to specified depth, multiple times (e.g. 10) -> ***rarefied.biom**
6. For each rarefied ***rarefied.biom** table, generate pairwise Unifrac (weighted and unweighted) distance matrices using ***treefile.tre*** -> ***betadiversity.txt**

STATISTICAL ANALYSES

For each ***betadiversity.txt** file we performed the same statistical analyses as described in the main text. Namely, PERMANOVA analyses of both weighted, and unweighted Unifrac distance matrices with time, treatment, and experimental block as factors (using the `adonis()` function in the vegan package (ref)).

Results & Discussion

Results are presented below in Figures 1 and 2. To summarize: for both DNA and RNA-based analyses, inference based on the weighted Unifrac metric is mostly insensitive to the choice of cutoff. Only in the case of the *P*-value associated with the Treatment effect for RNA-based analyses, is a consistent relationship between inference and cutoff choice evident (P-values become less significant with higher cutoff values). In contrast, analyses using the unweighted (i.e. presence-absence based) Unifrac metrics are highly sensitive to the choice of cutoff. For *P* values, there is also a very strong within-cutoff variation, apparently due to the sampling effects between rarefactions.

We do not claim that this sensitivity analysis represents the definitive solution of the “abundance cutoff” problem, but for the purposes of the study we describe in the main article, we propose that focusing our analysis on tests using the weighted Unifrac metric is the most pragmatic choice.

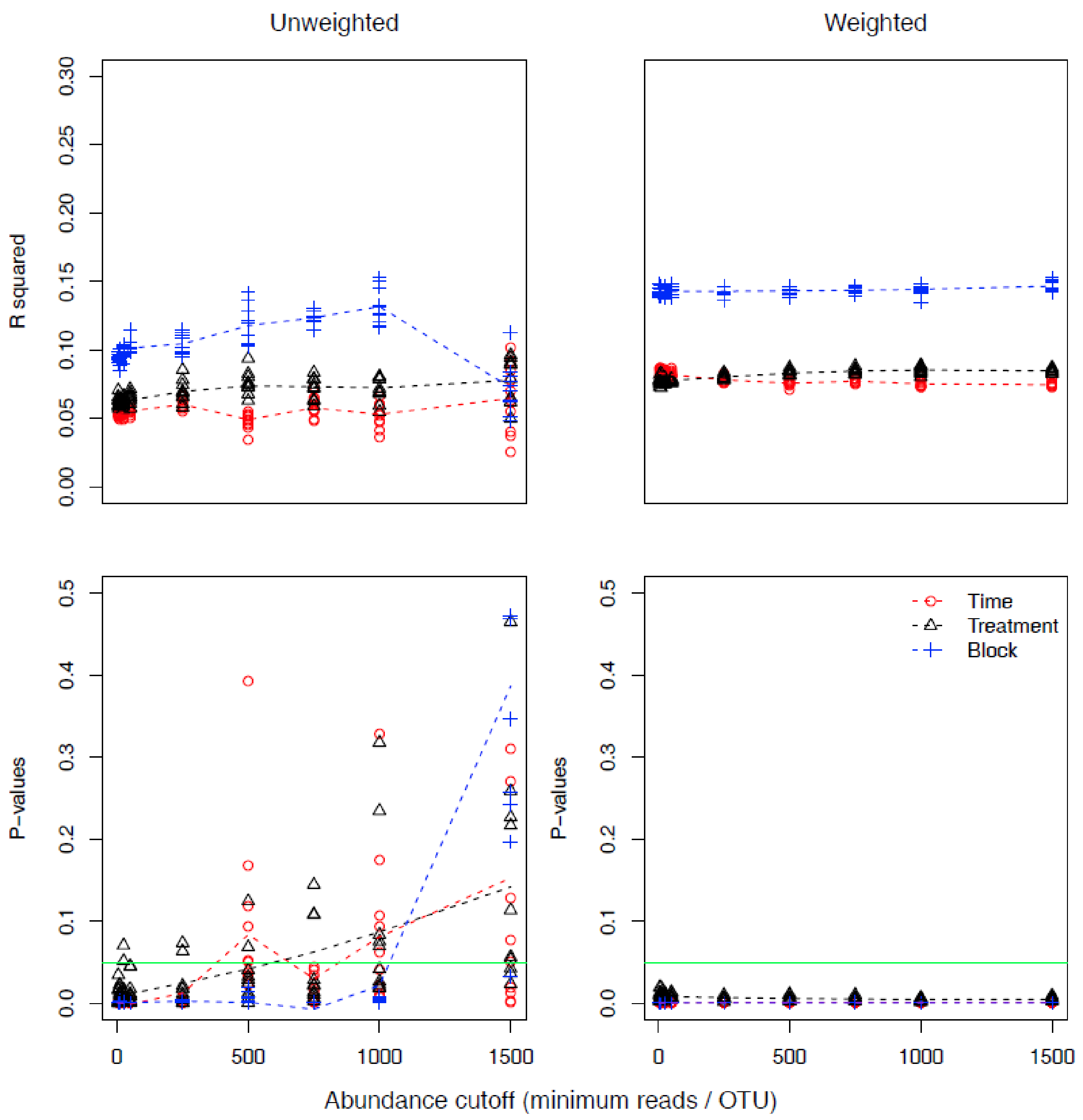

Figure 1 Effects of varying the choice of abundance cutoff (number *n* such that only OTUs with observed reads > *n* are retained in the dataset) on test statistics and inference in permutational MANOVA tests on DNA-based data. Each cutoff was used to generate a subset of a master OTU table, and then subject to 10 rarefactions and identifical model fits (see text).

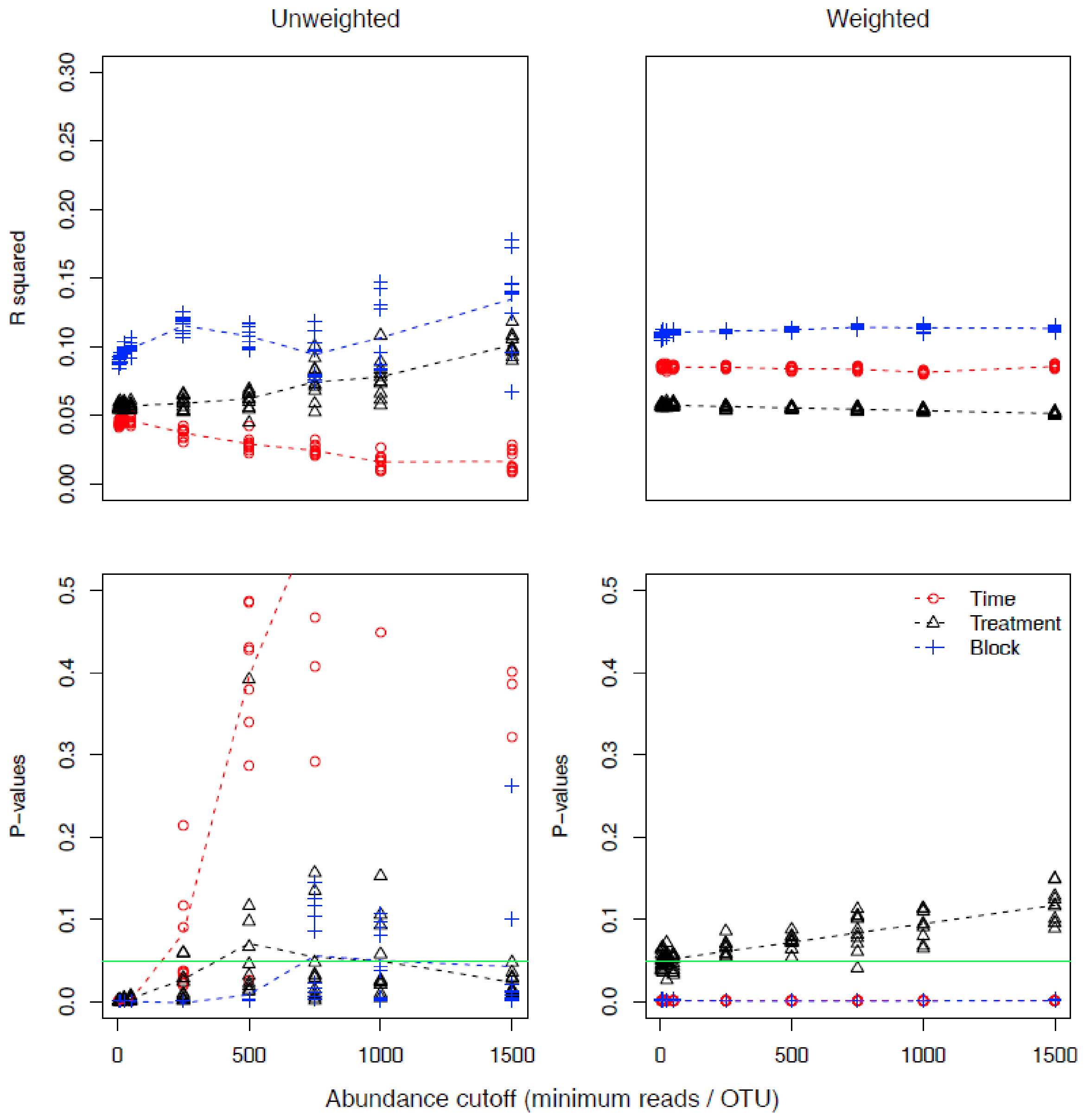

Figure 2 Data as for Figure 1, but using RNA-based amplicon profiles.
