# Supplementary material for: Compositional Stability of the Bacterial Community in a Climate-Sensitive Sub-Arctic Peatland
Source: Front Microbiol. 2017 Mar 7;8:317. doi: 10.3389/fmicb.2017.00317 (PMC5339224; doi:10.3389/fmicb.2017.00317)
Supplement: Supplementary file 3 [file Image_1.PDF]

Figure S1 *Read number distributions*

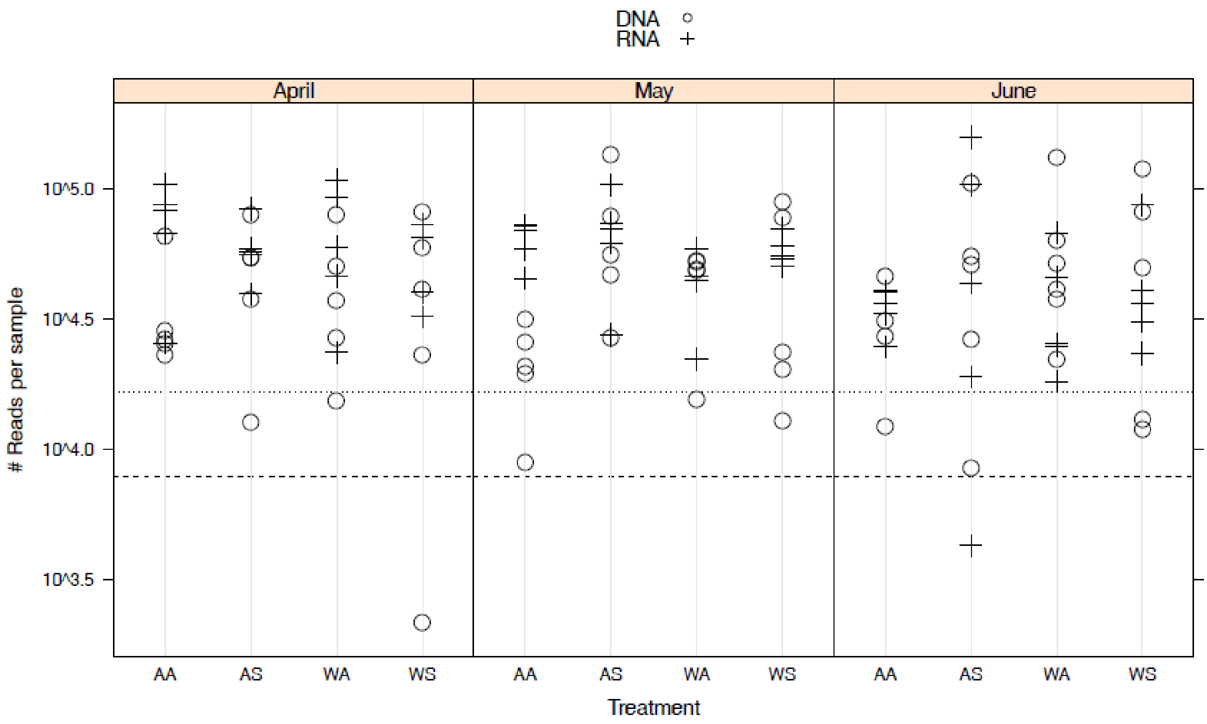

Figure S1 Distribution of reads per sample after quality filtering. Samples are plotted according to sampling time (April, May or June 2011) and climate change treatment (see main text for explanation of abbreviations). Open circles = DNA derived samples, crosses = RNA derived samples. The dotted and dashed lines show the rarefaction level for RNA-derived and DNA-derived samples respectively. Not log scale on the Y – axis.
