# Supplementary material for: Compositional Stability of the Bacterial Community in a Climate-Sensitive Sub-Arctic Peatland
Source: Front Microbiol. 2017 Mar 7;8:317. doi: 10.3389/fmicb.2017.00317 (PMC5339224; doi:10.3389/fmicb.2017.00317)
Supplement: Supplementary file 4 [file Image_2.PDF]

Figure S2 *Technical replicates*

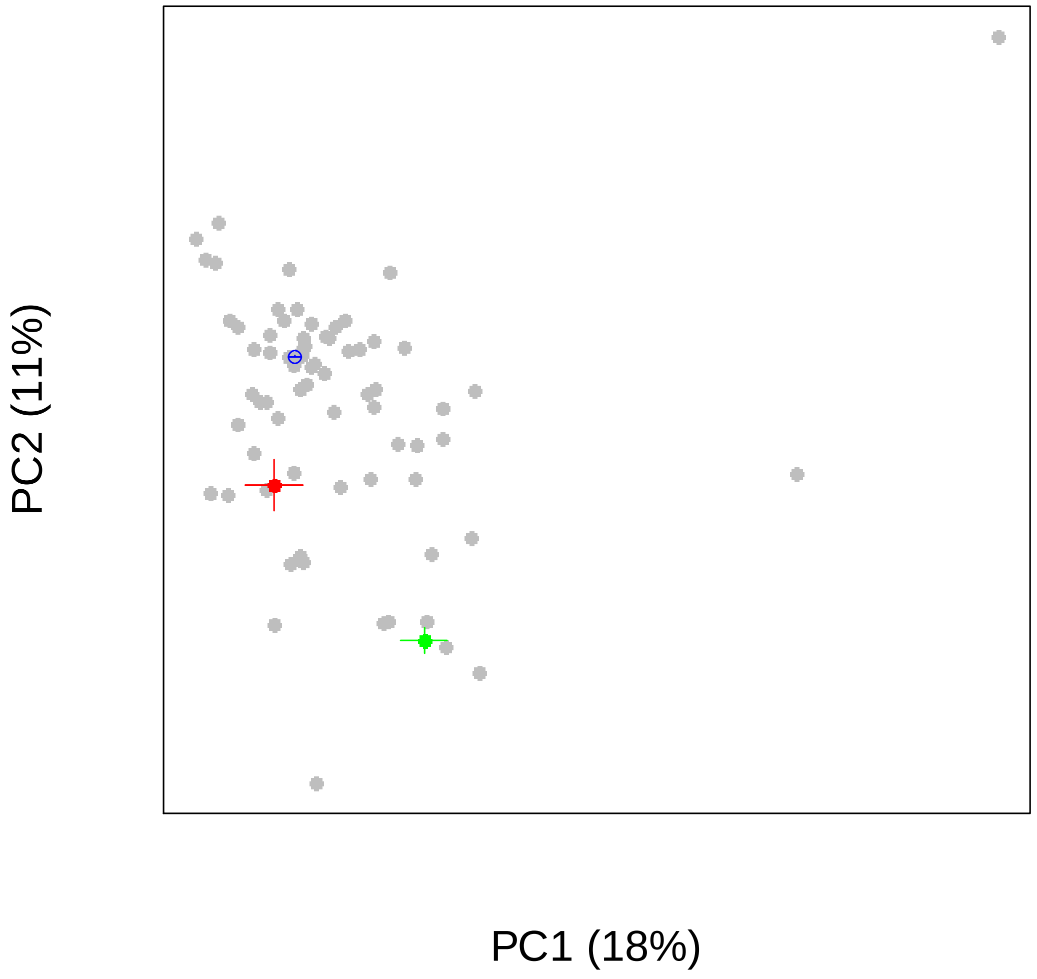

Figure S2 Principal coordinates analysis ordination of RNA-based community profiles. Coloured symbols and associated bars show mean and standard error of multiple technical replicates (same starting material, different PCR reaction and/or sequencing run). Number of replicates (red = 5; green = 4; blue = 2).
